# Forensic Autopsy in a Long-Term Survivor of Abusive Head Trauma: An Unusual Case Report

**DOI:** 10.3390/diagnostics15243176

**Published:** 2025-12-12

**Authors:** Jessika Camatti, Maria Paola Bonasoni, Rossana Cecchi, Anna Laura Santunione, Giancarlo Gargano, Giorgio Gualandri, Erjon Radheshi

**Affiliations:** 1 Department of Medicine and Surgery, University of Parma, Via Università 12, 43121 Parma, Italy; jessika.camatti@unipr.it; 2 Pathology Unit, Azienda USL-IRCCS di Reggio Emilia, Via Amendola 2, 42122 Reggio Emilia, Italy; 3 Unit of Legal Medicine, Department of Medical and Surgical Sciences, University of Bologna, Via Irnerio 49, 40126 Bologna, Italy; 4 Department of Biomedical, Metabolic and Neural Sciences, University of Modena and Reggio Emilia, Via Campi 287, 41125 Modena, Italy; rossana.cecchi@unimore.it (R.C.); annalaura.santunione@unimore.it (A.L.S.); 5 Neonatal Intensive Care Unit, Azienda USL-IRCCS di Reggio Emilia, Via Amendola 2, 42122 Reggio Emilia, Italy; giancarlo.gargano@ausl.re.it; 6 Unit of Legal Medicine and Bioethics, Azienda USL IRCCS di Reggio Emilia, Via Amendola 2, 42122 Reggio Emilia, Italy; giorgio.gualandri@ausl.re.it (G.G.); erjon.radheshi@ausl.re.it (E.R.)

**Keywords:** abusive head trauma, shaken baby syndrome, child abuse, forensic pathology, forensic autopsy, long-term outcomes

## Abstract

**Background and Clinical Significance:** Abusive head trauma (AHT), formerly known as Shaken Baby Syndrome (SBS), is a major cause of morbidity and mortality in infants and young children. While the forensic aspects of acute AHT are well described, autopsy findings in long-term survivors who die later in life remain poorly documented. **Case Presentation:** We describe a male infant diagnosed with AHT at 2 months of age, who survived with severe neurological sequelae until 19 years. He developed tetraparesis and epileptic encephalopathy, and ultimately died during hospitalization for bilateral pneumonia. Forensic autopsy revealed bilateral acute exudative pneumonia with respiratory failure. Neuropathological examination documented extensive chronic encephalomalacia, gliosis, and microcalcifications consistent with longstanding sequelae of AHT. These findings supported a causal nexus between the abusive head trauma sustained in infancy and the fatal pulmonary complication in adolescence. **Conclusions:** This case highlights the importance of forensic autopsy in long-term survivors of AHT. Establishing the causal relationship between early abusive injuries and late mortality is essential for both medical understanding and medicolegal evaluation.

## 1. Introduction

Shaken Baby Syndrome (SBS) was for several decades the prevailing medical term used to describe head trauma or traumatic brain injury inflicted on infants and young children. In recent years, the term Abusive Head Trauma (AHT) has been recommended by the American Academy of Pediatrics and the Centers for Disease Control and Prevention, as it better reflects the broad spectrum of abusive mechanisms involved, including shaking, blunt impact, and combined forces [1,2]. AHT typically affects children under five years of age and is characterized by subdural hematoma, retinal hemorrhages, and encephalopathy resulting from violent acceleration–deceleration forces [3]. Clinical manifestations range from mild, non-specific symptoms to acute life-threatening conditions, and survivors often suffer severe long-term sequelae, including epilepsy, motor disabilities, and cognitive impairments [4,5]. Reported mortality rates range between 10–20%, particularly in infants between 12 and 23 months of age [6]. The role of clinical and forensic investigation in acute AHT is well established [7,8,9].

Despite its widespread use in clinical and forensic practice, the diagnosis of AHT remains controversial in part of the scientific literature. The classical triad of subdural hematoma, retinal hemorrhages, and encephalopathy lacks absolute specificity and may occur in a variety of traumatic and non-traumatic conditions. Moreover, no universally accepted gold standard exists for confirming AHT, and multidisciplinary evaluation with careful exclusion of plausible alternative explanations is considered essential. These diagnostic uncertainties warrant explicit discussion when interpreting individual cases and discussing their medicolegal implications [1,2,3,4,5,6,7,8,9].

Children with AHT may present with clear signs of trauma or abuse (such as unexplained bruising, fractures, seizures, or loss of consciousness), but many also have nonspecific symptoms like vomiting and excessive fussiness. A thorough physical examination—including assessment of head circumference, skin, oral cavity, and ears—is recommended to identify children who may benefit from further evaluation for abuse. The American College of Surgeons’ Advanced Trauma Life Support (ATLS) methodology is endorsed for initial evaluation and stabilization, including primary and secondary surveys focused on airway, breathing, circulation, neurologic disability, and exposure, with special attention to cervical spine stabilization and neurologic assessment (e.g., pupillary response, Glasgow Coma Scale) [7]. Physical findings in AHT are often variable and subtle. Bruising is a key sentinel injury in infants under 12 months, and its recognition is critical for preventing escalation to more severe abuse. Sentinel injuries, especially bruising, are frequently missed or underappreciated in cases later confirmed as definite abuse [7]. Chronic presentations may include macrocephaly, seizures, sun-setting eyes, lethargy, and irritability, with some children presenting with occipitofrontal circumference above the 90th percentile due to evolving chronic subdural collections [7].

By and large, the American Academy of Pediatrics and the American Academy of Ophthalmology emphasize a high index of suspicion, thorough examination, and standardized trauma evaluation protocols for suspected AHT, with attention to both acute and chronic presentations and the importance of recognizing subtle sentinel injuries [7].

However, there is limited knowledge on forensic autopsy findings in long-term survivors who subsequently die from complications related to the initial abusive injury. Understanding such cases is essential both for medical interpretation and for establishing the causal nexus in legal contexts [10]. Here, we report the case of a male patient diagnosed with AHT at 2 months of age, who survived with severe neurological impairment until 19 years of age, when he died from bilateral pneumonia. A full forensic autopsy, including histological examination, was performed to determine the cause of death and its relationship to the initial abusive trauma.

## 2. Case Report

A 2-month-old male infant was admitted to the Pediatric Emergency Department in a state of torpor, with pale skin, poor peripheral circulation, basal hypotonia, hyperexcitability, high-pitched crying, tense anterior fontanelle, and hypothermia. During hospitalization in the Neonatology Unit, the patient developed recurrent seizures. Lumbar puncture revealed blood-tinged cerebrospinal fluid, while electroencephalography documented status epilepticus involving the left hemisphere. Brain CT and MRI demonstrated diffuse ischemic edema, a bilateral subdural hematoma, subarachnoid hemorrhage, fractures of the left parietal and occipital bones, and multiple intraparenchymal cavitations. Cranial ultrasound confirmed severe hypoxic–ischemic encephalopathy. Ophthalmologic evaluation revealed extensive retinal, pre-retinal, and vitreal hemorrhages.

The patient was discharged after 30 days with a diagnosis of status epilepticus, bilateral skull fractures, and retinal hemorrhage, and referred to Child Neuropsychiatry services. Follow-up at 5 months of age showed severe neuromotor impairment consistent with chronic sequelae of craniocerebral trauma. Brain MRI revealed multicystic encephalopathy involving the right cerebral hemisphere and left frontoparietal regions, with sparing of basal nuclei, thalamus, and cerebellum. In the absence of natural disease and considering the clinical, radiologic, and ophthalmologic findings, a diagnosis of AHT was established.

Importantly, the diagnosis was reached only after considering and excluding alternative plausible traumatic scenarios. At the time of the initial evaluation, no history of accidental trauma (e.g., road-traffic injury or high-energy impact) was provided, and the pattern and multiplicity of lesions—including bilateral skull fractures, extensive retinal hemorrhages, and diffuse hypoxic–ischemic injury—were assessed as incompatible with minor accidental mechanisms. Thus, the diagnosis reflected the combination of findings and the lack of any reasonable accidental explanation.

Legal proceedings followed, and the father was found guilty of child abuse when the child was 5 years old. During the trial, he never described how the severe trauma was caused. However, the skull fractures suggested battering against a rigid surface. The child was placed in the exclusive care of his mother. Despite profound neurological impairment, the patient remained clinically stable until the age of 18 years, with good medical control of seizures (levetiracetam 1000 mg/die and depakin 600 mg/die), although many episodes of dysphagia were reported despite a creamy, semi-liquid diet. The patient was then found in bed with tonic–clonic seizures and therefore hospitalized. Thoracic X-rays and CT scan showed bilateral pneumonia. Diagnosis of aspiration pneumonia with superinfection was confirmed with isolation of Klebsiella Pneumoniae and Pseudomonas Aeruginosa within the bronchioalveolar lavage (BAL). CT imaging documented extensive encephalomalacia with ventricular enlargement, and EEG showed diffuse slowing of background activity with no epileptiform discharges. The patient was then discharged in a specific facility for disabled people, in good condition, with the recommendations of continuing a creamy, semi-liquid diet. A midline and a PICC (peripherally inserted central catheter) were placed for medications and parenteral nutrition. Multiple dysphagic episodes were reported.

After one year, at 19 years of age, the patient was hospitalized again with fever, psychomotor agitation, tetraparesis, and epileptic encephalopathy. Bilateral pneumonia was confirmed with thoracic X-rays. He died during hospitalization due to progressive respiratory failure. At the request of the Public Prosecutor, a forensic autopsy was performed to clarify the cause of death and assess its causal relationship to the antecedent AHT. The autopsy was performed four days after the patient’s death.

### 2.1. External Examination and Autopsy

The body measured 149 cm in height and weighed 28 kg. There was marked muscle wasting and poor trophic condition. No recent traumatic injuries were detected. At autopsy, bilateral pleural effusion (200 mL each side) was present.

The lungs (right: 350 g, left: 350 g) were reduced in volume, with pinkish-gray coloration in the apico-marginal regions and purplish-red discoloration in the basal areas. The bronchial tree was patent and lined with mucus. The vascular tree was patent and contained fluid blood. On sectioning, the pulmonary parenchyma appeared congested and exuded abundant edema fluid. Considering the radiological diagnosis of pneumonia, microbiological cultures were not carried out. Multiple areas of dark, friable parenchymal consolidation were identified in both lungs and sampled for histological analysis.

**Figure 1:**
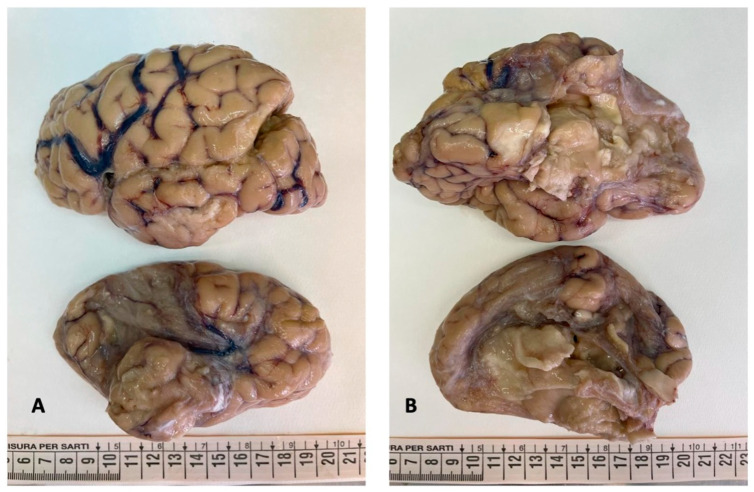
Brain gross examination: both cerebral hemispheres showed meningeal congestion, particularly severe on the left ((**A**,**B**), top figure). The right hemisphere presented cystic leukomalacia on the frontal, parietal, temporal, and insular regions ((**A**,**B**), bottom figure).

**Figure 2:**
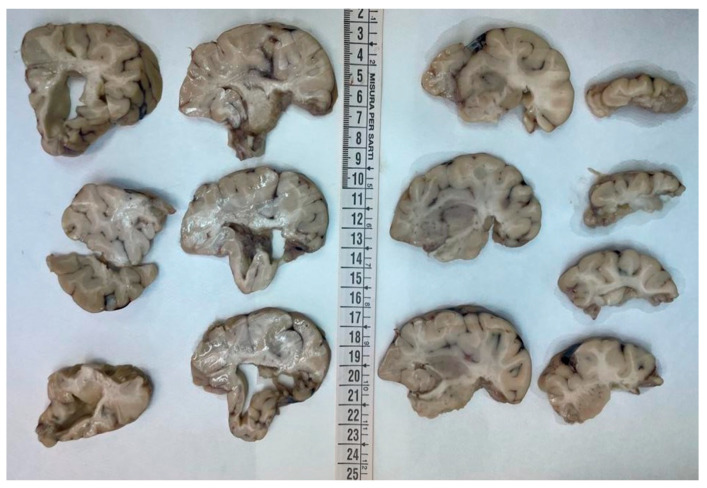
Coronal sections of the left hemisphere: the cortex was diffusely atrophic with shrinkage of the white matter and the ventricular system was overall enlarged.

**Figure 3:**
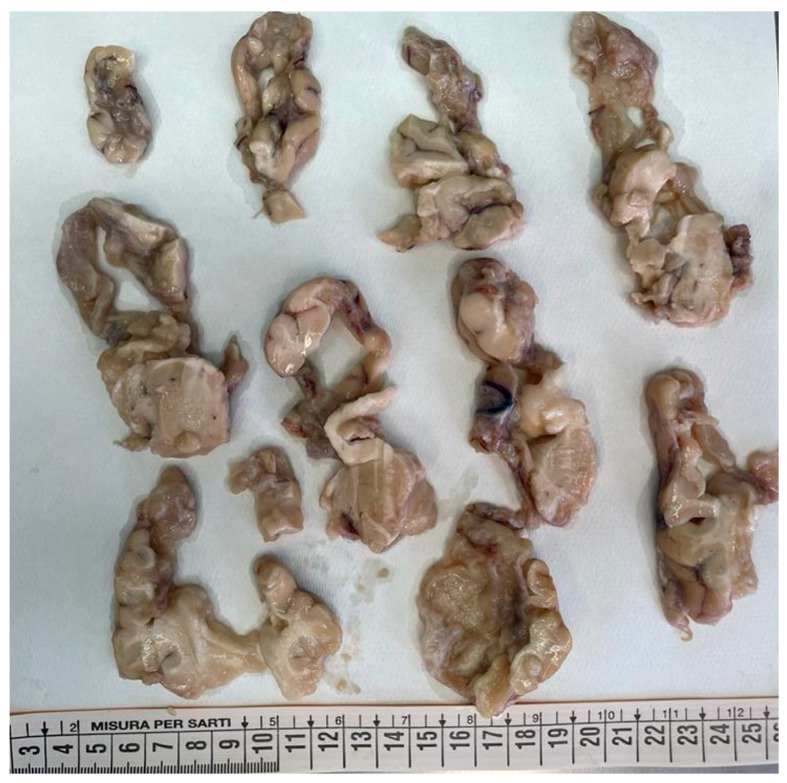
Coronal sections of the right hemisphere: the tissue was extremely friable. However cortical and subcortical cystic malacia was evident in the frontal, parietal, temporal, and insular regions (from top to bottom).

The brain was completely removed and fixed in formalin for one month prior to examination. Brain pathology showed marked congestion of meningeal vessels, especially on the left side. The right cerebral hemisphere presented widespread cortical-subcortical malacic areas in the frontal, parietal, temporal, and insular regions. The left hemisphere exhibited frontoparietal cortical-subcortical malacic lesions. There was diffuse dilation of the entire ventricular system (Figure 1, Figure 2 and Figure 3).

A sample of blood was frozen and preserved for potential further analysis. However, toxicology was not requested as not relevant, due to the patient’s recent clinical history.

### 2.2. Histology

Histological sampling included:

- 3 samples for each pulmonary lobe (9 from the right lung, 6 from the left) lung;
- 2 samples for each cerebral lobe for both hemispheres (frontal, parietal, temporal occipital lobes) including the cortex and the white matter; basal ganglia, thalamus, and hippocampus were sampled from each cerebral side; 2 samples from the cerebellar vermis and 2 samples for each cerebellar hemisphere, including the cortex, the white matter, and the gray nuclei; and the encephalic trunk was thoroughly sampled (mesencephalon, pons, and medulla);
- One sample for each organ (thyroid, heart, liver, adrenal glands, and kidneys).

**Figure 4:**
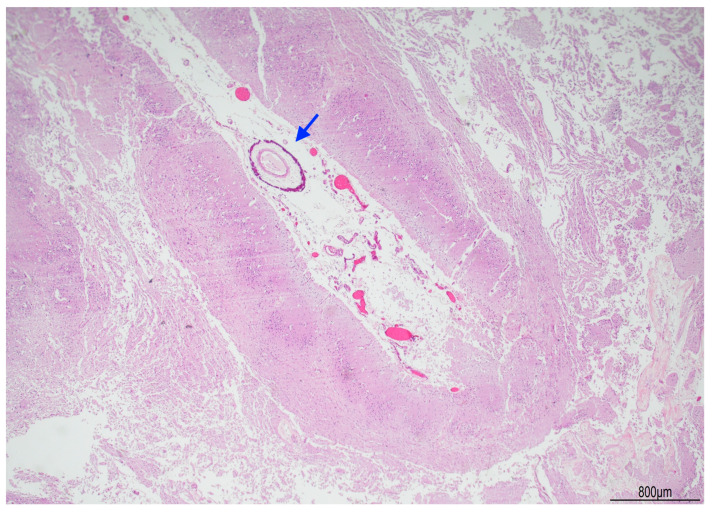
Right brain cortex: cystic leukomalacia was prominent with cortical atrophy and disrupted white matter. The blue arrow indicates a meningeal vessel showing sub-adventitial calcification (Hematoxylin and Eosin, 2 HPF).

**Figure 5:**
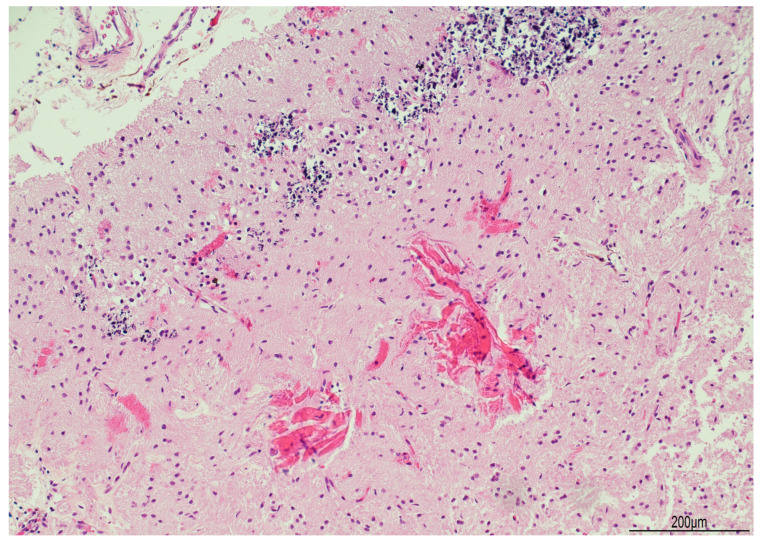
Ferruginated neurons: the cerebral cortex was atrophic with widespread neuronal ferrugination (top). Proteinaceous eosinophilic deposition (Rosenthal fibers) was also seen in the white matter (bottom) (Hematoxylin and Eosin, 10 HPF).

**Figure 6:**
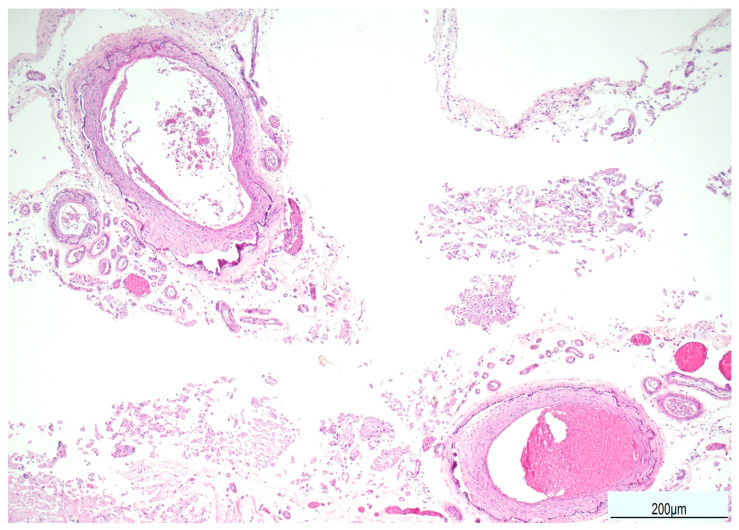
Calcification of the external elastic lamina of the meningeal arteries (Hematoxylin and Eosin, 10 HPF).

Tissue samples were formalin-fixed, paraffin-embedded, and stained with hematoxylin–eosin. Brain samples were very soft and most of the sections were disrupted after tissue processing. The brainstem showed neuronal ischemic changes, while the cerebellum showed ischemic features with Purkinje cell loss. The brain documented diffuse cortical and subcortical leukomalacia with neuronal loss, isomorphic and anisomorphic gliosis with reactive astrocytes and gemistocytes, Rosenthal fibers, and ferruginated residual neurons (Figure 4 and Figure 5). The meningeal arteries presented sub-adventitial mineralization of the tunica media and calcification of the external elastic lamina (Figure 6). Scattered microcalcifications were also found in the cerebral parenchyma as well as mineralization of the small and medium vessels. Special stains were not carried out as the histological findings were sufficiently informative for interpretation.

**Figure 7:**
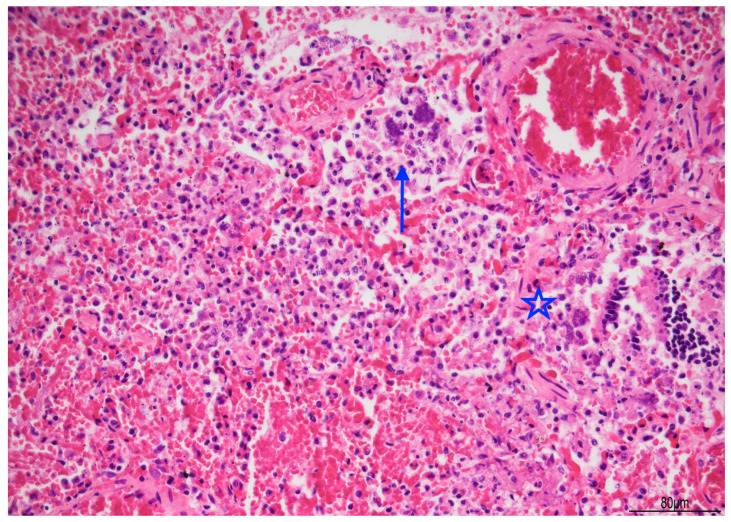
Acute pneumonia: alveoli were filled with neutrophils and bacteria (blue arrow). Bacteria were also found in bronchioles (star). Abundant erythrocytes within the alveoli were also present (Hematoxylin and Eosin, 20 HPF).

**Figure 8:**
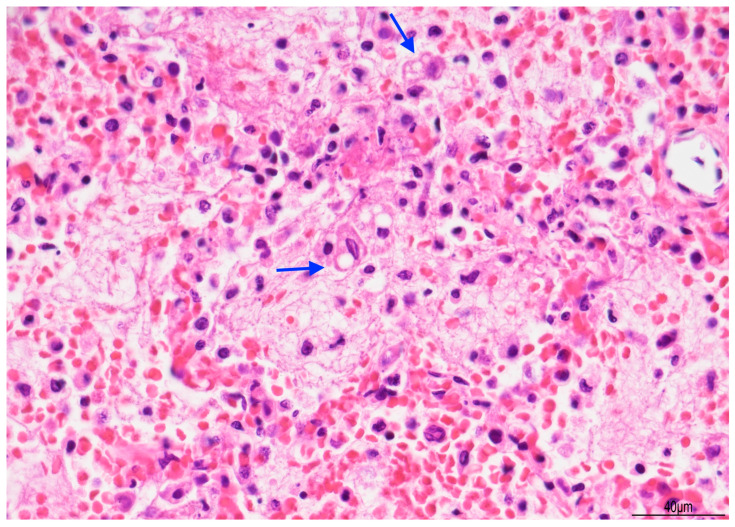
Exudative pneumonia: abundant alveolar fibrin was intermixed with lipid-laden macrophages, compatible with overlapping early lipoid pneumonia (blue arrows) (Hematoxylin and Eosin, 10 HPF).

**Figure 9:**
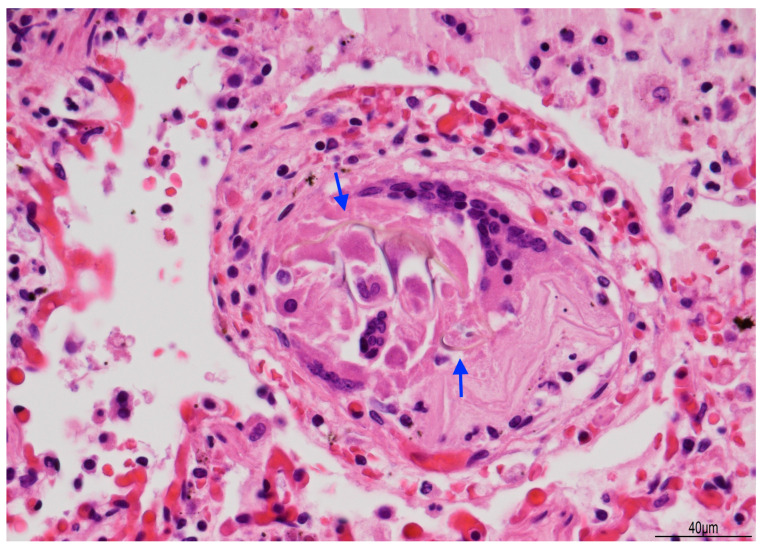
Giant cell reaction to foreign body: brown fibers (arrows), likely due to aspirated material, were seen within multinucleated giant cells (Hematoxylin and Eosin, 40 HPF).

Lung histology revealed acute exudative pneumonia with abundant neutrophils, fibrin deposition, intra-alveolar hemorrhage, bacterial colonies (cocci), abundant lipid-laden macrophages (compatible with early lipoid pneumonia), and focal foreign-body giant cell reaction to aspirated material (Figure 7, Figure 8 and Figure 9). Histochemistry for PAS, PAS-D, and Grocott were performed to rule out fungal colonization and resulted negative.

### 2.3. Cause of Death

The cause of death was determined as bilateral acute exudative pneumonia due to prolonged micro-aspiration and superinfection with subsequent respiratory failure, in a patient with tetraparesis and epileptic encephalopathy as sequelae of AHT. Considering the previous episodes of aspiration pneumonia and superinfection, associated with prolonged immobility, hospital and/or community pneumonia were therefore ruled out.

The forensic investigation confirmed the causal relationship between the initial AHT and the subject’s long-term disability, which predisposed to the fatal outcome. Accordingly, the father was found guilty of his son’s death; the guilty verdict occurred approximately two years after the fatal event.

## 3. Discussion

Abusive head trauma (AHT) remains a leading cause of death and disability in early childhood and is associated with a broad spectrum of long-term neurological and developmental sequelae. The present case—death at 19 years after infancy-onset AHT with severe chronic encephalopathy—offers a rare window into the forensic implications of delayed mortality after AHT and underscores how early abusive injury can set in motion a chain of events that culminates in a late fatal outcome.

### 3.1. Long-Term Outcomes After AHT

Across cohorts, long-term disability after AHT is common and often multifaceted. Claims-based studies and clinical series consistently report high rates of neurodevelopmental impairment by school age, with epilepsy, developmental and learning disorders, motor and visual deficits among the most frequent sequelae. In a national claim cohort, disability was present in 68% at 5 years and rose to 82% at 11 years, with a significant increase in impairment between ages 5 and 11, highlighting that morbidity accrues over time rather than plateauing after the acute phase [5]. Similar results were observed in a large administrative cohort with 72% of children demonstrating at least one disability by age 5, particularly developmental delays (47%), learning disorders (42%), and epilepsy (36%) [11].

Detailed follow-up studies corroborate this burden. In a rehabilitation cohort with a median 8-year follow-up, only 15% achieved a “good outcome,” while severe neurological impairment, attention and behavioral disorders, language abnormalities, and the need for ongoing rehabilitation were frequent [12]. Data from a middle-income setting similarly show substantial mortality (10%) and disability (≥35% among survivors), with multiple disabilities more common when children were ≥3 years at last review, underscoring the need for prolonged, multidisciplinary follow-up beyond early childhood [13]. A regional French series further documents the limited duration and fragmentation of long-term follow-up and a sizeable proportion of children with persistent neurological abnormalities and social care needs years after the index event [14]. Recent narrative synthesis confirms that more than half of patients have disabilities after discharge, with variability driven by heterogeneous measures and high attrition; nonetheless, the signal of long-term morbidity is strong and consistent across studies [15]. Our patient’s trajectory—multicystic encephalopathy, tetraparesis, epileptic encephalopathy, and lifelong dependence—is aligned with these data and illustrates how chronic disability predisposes to secondary complications (aspiration, recurrent infections), ultimately culminating in fatal bilateral pneumonia in adolescence.

### 3.2. Predictors of Mortality and Disability

Early injury severity and specific clinical/imaging markers are associated with worse outcomes. Features such as brainstem dysfunction, the need for mechanical ventilation, hypoxic–ischemic injury, and cerebral edema have been linked to higher mortality and later disability; neurological and visual impairment at discharge are also associated with poorer trajectories [13]. Broader syntheses highlight the prognostic value of lower GCS/motor scores, seizures, raised intracranial pressure, and systemic hypotension, emphasizing that both primary and secondary brain insults shape long-term outcome [15]. The extensive hypoxic–ischemic encephalopathy and diffuse encephalomalacia documented in our case are therefore consistent with a high-risk phenotype for severe long-term morbidity.

### 3.3. Imaging and Neuropathological Correlates

Neuroimaging is critical to characterize the pattern and extent of injuries in AHT. MRI commonly reveals subdural collections, diffuse hypoxic–ischemic injury, parenchymal lacerations, and cervicomedullary/spinal involvement, often in the relative absence of external signs [16]. The chronic neuropathological findings in our case—diffuse cortical–subcortical leukomalacia, gliosis, mineralization/microcalcifications, and ventriculomegaly—cohere with the expected end-stage substrate of remote severe AHT-related injury and provide an anatomic basis for refractory epilepsy, profound motor impairment, and dysphagia with aspiration risk.

### 3.4. Forensic and Medicolegal Causation

From a forensic perspective, this case illustrates the causal chain linking the early abusive injury to the terminal event decades later. As previously emphasized in medicolegal analyses, AHT can entail delayed yet progressive cognitive/behavioral and motor sequelae that only fully manifest as environmental demands increase, with substantial implications for care needs and legal redress [16]. In our case, the forensic autopsy established bilateral acute exudative pneumonia as the immediate cause of death and, critically, documented the permanent neurological condition (tetraparesis with epileptic encephalopathy) created by the index AHT. This supports a causal nexus whereby the antecedent abusive injury produced a stable pathologic state that predisposed to aspiration and recurrent infections, ultimately leading to fatal pneumonia. Indeed, documented patterns of infection in patients with stable predisposing conditions support the inference that a chronic vulnerability can precede a fatal infectious outcome [17]. Recent autopsy-based reports similarly highlight the value of full morphological documentation in infection-related deaths with complex premorbid contexts, ensuring that the terminal pathway is anatomically substantiated [18].

Generally speaking, forensic and pulmonary pathology criteria for aspiration are not standardized, but consensus in the medical literature defines aspiration-related pulmonary injury by the presence of foreign material (food, gastric contents, or other debris) in the airways or alveoli, often accompanied by acute or chronic inflammatory changes, and sometimes bacterial colonization. Forensic pathologists rely on histopathologic evidence of aspirated material, associated bronchopneumonia, and clinical context (e.g., impaired consciousness, neurodisability) to support a diagnosis of aspiration as a contributing factor in death. The American College of Chest Physicians and recent reviews emphasize that aspiration syndromes are classified by the nature and volume of aspirated material, with macroaspiration (large-volume) being the sine qua non for aspiration pneumonia, and microaspiration contributing to other pulmonary syndromes. Pulmonary findings may include chemical pneumonitis, bacterial pneumonia, or foreign body reaction, with acute lung injury and hypoxemia as common sequelae [19,20,21,22]

Mortality and respiratory complication literature in severe neurodisability demonstrates that individuals with intellectual disability and severe neurological impairment have a markedly increased risk of respiratory-associated death, particularly from pneumonia and aspiration events. Meta-analyses report standardized mortality ratios for respiratory deaths in intellectual disability populations as high as 10.86 compared to the general population, with pneumonia being a leading cause. Risk factors include severity of disability, comorbid neurological disorders, and impaired airway protection [23,24]. The American College of Chest Physicians recommends proactive respiratory management in neuromuscular weakness to mitigate these risks [22].

Comparable autopsy case reports of late death after infant abusive head trauma are not identified in the medical literature after a targeted search. The American Academy of Pediatrics and American Academy of Ophthalmology technical report notes that autopsy series of abusive head trauma (AHT) are generally small, descriptive, and lack robust controls, with most deaths occurring acutely or subacutely. To the best of our knowledge, in the current literature there is no systematic documentation of late deaths (months to years post-injury) in infants with prior AHT [7,25].

Our report is limited by its single-case design, which restricts the generalizability of the findings. Microbiological studies were not pursued as the clinical history was clear enough to indicate pneumonia. Aspiration pneumonia with superinfection was inferred from the histological findings. However, microbiological cultures would have been useful to better characterize the infectious process by identifying the specific pathogens involved. Finally, interpretations regarding causation reflect the medicolegal standards of the authors’ jurisdiction and may not be directly applicable in regions with differing legal frameworks for attributing remote injuries to later mortality.

### 3.5. Diagnostic Controversies and Implications

The diagnosis of AHT is widely applied in pediatrics and forensic medicine, yet it remains the subject of debate, particularly when based solely on the combination of subdural hemorrhage, retinal hemorrhages, and encephalopathy. Critics have pointed out limitations in the evidence supporting the specificity of this triad, as well as the lack of a true gold standard for confirming abuse. Consequently, in cases lacking external injuries or fractures, alternative explanations—such as accidental trauma, underlying medical conditions, or perinatal factors—must be rigorously evaluated.

Although current pediatric and forensic practice recognizes AHT as a clinicopathological diagnosis that integrates clinical history, imaging, and ophthalmologic findings, debate persists in the literature regarding the diagnostic specificity of classical constellations (e.g., subdural hemorrhage, retinal hemorrhage, encephalopathy) and the quality of evidence underpinning some inferences. Recent commentary has argued that parts of the evidence base remain methodologically limited, with concerns about circular reasoning and uncertain error rates in diagnostic pathways [26]. The social context is a critical determinant in the risk and occurrence of AHT in children. Social factors such as household composition, socioeconomic deprivation, intimate partner violence, substance abuse, prior police involvement, and adverse childhood experiences are strongly associated with increased risk of AHT [27]. Perpetrators are most commonly male caregivers, and risk is elevated in settings of young maternal age, late prenatal care, low income, and crowded households [7]. Household composition also influences perpetrator identity: infants with young siblings are more likely to be injured by biological parents, while those without siblings or who are older are more likely to be injured by non-parental caregivers such as a parent’s partner [28]. The presence of substance abuse, unknown number of adults in the home, and prior police involvement further increase risk [27]. However, reported ‘risk factors’ for AHT must be interpreted cautiously, as they derive from cohorts in which the diagnostic criteria lack a definitive gold standard. These epidemiological patterns therefore reflect associations within clinically defined AHT populations, rather than etiologic determinants with proven specificity.

In parallel, legal scholarship has questioned the handling of expert testimony in court and emphasized the need for impartial, methodologically sound expert opinions, especially in contested SBS/AHT cases [29]. These debates do not diminish the significance of careful forensic documentation in individual cases; rather, they underscore the importance of transparent methodology, comprehensive differential diagnosis, and multidisciplinary corroboration.

In the present case, however, the diagnostic uncertainty typically described in the literature was mitigated by the presence of multiple unequivocal signs of high-energy trauma, including bilateral cranial fractures and extensive retinal, subdural, and parenchymal injuries. The constellation, severity, and distribution of lesions rendered accidental mechanisms (e.g., household falls or even road-traffic accidents) implausible in the clinical context available at the time. This combination of findings, together with the exclusion of alternative causes, strengthens the inference of abusive etiology and its long-term consequences.

### 3.6. Public Health and Follow-Up

The high frequency of accumulating disability after AHT mandates long-term, coordinated follow-up, spanning neurology, rehabilitation, nutrition/swallowing, ophthalmology, and psychosocial support. Cohorts from both high- and middle-income settings highlight substantial care needs, attrition in follow-up, and significant social impact (e.g., foster placement, special education). Contemporary reviews recommend standardized assessment tools, structured developmental surveillance, and attention to social determinants (e.g., insurance/coverage) that influence access to services and outcomes.

## 4. Conclusions

This case contributes rare forensic autopsy documentation of a long-term survivor of AHT with delayed fatality, integrating clinical history, imaging, and neuropathology to demonstrate the enduring causal link between the abusive injury and death. Its alignment with multi-cohort evidence strengthens the inference that severe early AHT can establish a chronic state that predisposes to lethal complications years later. Forensic autopsy in such contexts is pivotal to clarify causation, inform death certification, and support medicolegal evaluation.

This case also underscores the importance of transparent diagnostic reasoning in AHT, acknowledging both the lack of a universal gold standard and the need to exclude plausible alternative mechanisms. In cases with unequivocal traumatic findings—as in the present report—multidisciplinary clinical evaluation combined with forensic documentation provides robust support for the diagnosis and its long-term consequences.

In summary, AHT is not only an acute pediatric emergency but a condition with lifelong ramifications. Survivors frequently accrue new deficits over time, and severe chronic disability can indirectly lead to late fatal outcomes. Comprehensive forensic autopsy remains essential to document the pathological continuum from early abusive injury to delayed death and to ground medicolegal conclusions in objective anatomic evidence.

## Data Availability

The data presented in this study are available on request from the corresponding author due to privacy.

## Abbreviations

The following abbreviations are used in this manuscript:

AHT	Abusive Head Trauma
SBS	Shaken Baby Syndrome
